# Vitamin D and reproductive disorders: a comprehensive review with a focus on endometriosis

**DOI:** 10.1186/s12978-024-01797-y

**Published:** 2024-05-02

**Authors:** Pooya Farhangnia, Morvarid Noormohammadi, Ali-Akbar Delbandi

**Affiliations:** 1 Department of Immunology, School of Medicine, Iran University of Medical Sciences, Tehran, Iran; 2 Immunology Research Center, Institute of Immunology and Infectious Diseases, Iran University of Medical Sciences, Tehran, Iran; 3 Immunology Board for Transplantation and Cell-Based Therapeutics (ImmunoTACT), Universal Scientific Education and Research Network (USERN), Tehran, Iran; 4 Department of Nutrition, School of Public Health, Iran University of Medical Sciences, Tehran, Iran; 5 Reproductive Sciences and Technology Research Center, Department of Immunology, School of Medicine, Iran University of Medical Sciences, Tehran, Iran

**Keywords:** Endometriosis, Vitamin D, Vitamin D deficiency, Vitamin D binding protein, Reproductive disorders

## Abstract

Vitamin D is a fat-soluble steroid hormone that was initially known only for regulating calcium and phosphorus levels and maintaining bone health. However, it was later discovered that many organs express vitamin D metabolizing enzymes and have a ligand for vitamin D, which regulates the expression of an extensive assortment of genes. As a result, vitamin D is indispensable for the proper function of organs, and its deficiency is believed to be a critical factor in symptoms and disorders such as cardiovascular diseases, autoimmune diseases, and cancers. The significance of vitamin D in reproductive tissues was recognized later, and studies have revealed its crucial role in male and female fertility, as well as proper reproductive function during pregnancy. Vitamin D deficiency has been identified as a risk factor for infertility, gonadal cancers, pregnancy complications, polycystic ovary syndrome, and endometriosis. However, data investigating the association between vitamin D levels and reproductive disorders, including endometriosis, have encountered inconsistencies. Therefore, the present study aims to review existing research on the effect of vitamin D on proper reproductive function, and the role of deficiency in reproductive diseases and specifically focuses on endometriosis.

## Introduction

Vitamin D is a fat-soluble substances that plays a vital role in maintaining good health. Calcitriol or 1,25(OH)2D3, is the biologically active form of vitamin D. Mechanistically, vitamin D fulfils its non-genomic role by binding to the cellular membrane vitamin D receptor (VDR), while its genomic role involves binding to the cytoplasmic VDR [1]. VDRs can be found all throughout the human body [2]. VDR is present in various cells within the male reproductive system, including ducts, sertoli and leydig cells, germ cells, developing spermatozoa, and mature sperm. In females, VDR is found in the reproductive tract as well as in the uterus and ovary. The placenta also shows VDR expression during pregnancy [3].

Vitamin D plays a significant role in reproductive health. It is involved in many physiological reproductive processes, including ovarian steroidogenesis, folliculogenesis, spermatogenesis, and acrosome reaction. It is also correlated with sperm quality and ovarian reserve [4]. Vitamin D has been associated with conditions like polycystic ovarian syndrome (PCOS) and endometriosis [4, 5]. Observational studies have shown higher rates of preeclampsia, preterm birth, bacterial vaginosis, and gestational diabetes in women with low vitamin D levels [5]. Vitamin D deficiency has been linked with infertility [6, 7]. It is also likely that vitamin D is involved in the process of conception, implantation, and the development of the placenta [7–9]. Moreover, vitamin D is especially significant for a healthy pregnancy [10–13]. While these findings suggest a crucial role of vitamin D in reproductive health, it’s important to note that the evidence is still being researched, and the clinical implications are not fully understood yet [14].

Endometriosis is a medical condition wherein tissue resembling the lining of the uterus, known as endometrial tissue, is located outside the uterus. This condition leads to pelvic pain, dysmenorrhea, dyspareunia, and infertility, and it affects the quality of life for women [15–17]. The prevalence peak for endometriosis is between 25 and 35 years old, which falls within the reproductive age range. Environmental exposures and diet are among the risk factors associated with this condition [18]. Hormone therapies are commonly used as a first-line treatment for certain conditions, but they are often not curative and can lead to additional side effects [19].

Vitamin D has been identified for its potential in treating endometriotic lesions because of its anti-inflammatory and immunomodulatory properties. Our research has revealed that patients with endometriosis often have a deficiency in vitamin D [20]. Endometriosis shares some characteristics with cancer and may be associated with an increased risk of autoimmune disorders. Vitamin D is suggested as a potential factor in this process due to its anti-proliferative, anti-inflammatory, and immunomodulatory properties [21–23]. Additionally, certain immune cells express VDR, as well as vitamin D metabolizing enzymes such as 1-α hydroxylase and 24-hydroxylase [24–26]. Our studies have demonstrated a decrease in proliferation in peritoneal fluid mononuclear cells (PFMCs) and peripheral blood mononuclear cells (PBMCs) of patients with endometriosis after receiving vitamin D treatment [27]. Furthermore, our research has shown the effects of vitamin D3 on adhesion, invasion, infiltration, proliferation, cell death, cytokine production, and angiogenesis potential of eutopic and ectopic endometrial stromal cells from women with endometriosis [28].

The relationship between vitamin D and endometriosis is not fully understood, prompting a need for a review of the impact of vitamin D on reproductive health. The article will explore the significance of vitamin D in male and female reproductive organs, its role in infertility, and the potential association between vitamin D deficiency and reproductive disorders like endometriosis. A proposed framework for how vitamin D may affect reproductive tissues will also be discussed.

## Vitamin D

### Structure and metabolism

**Fig. 1:**
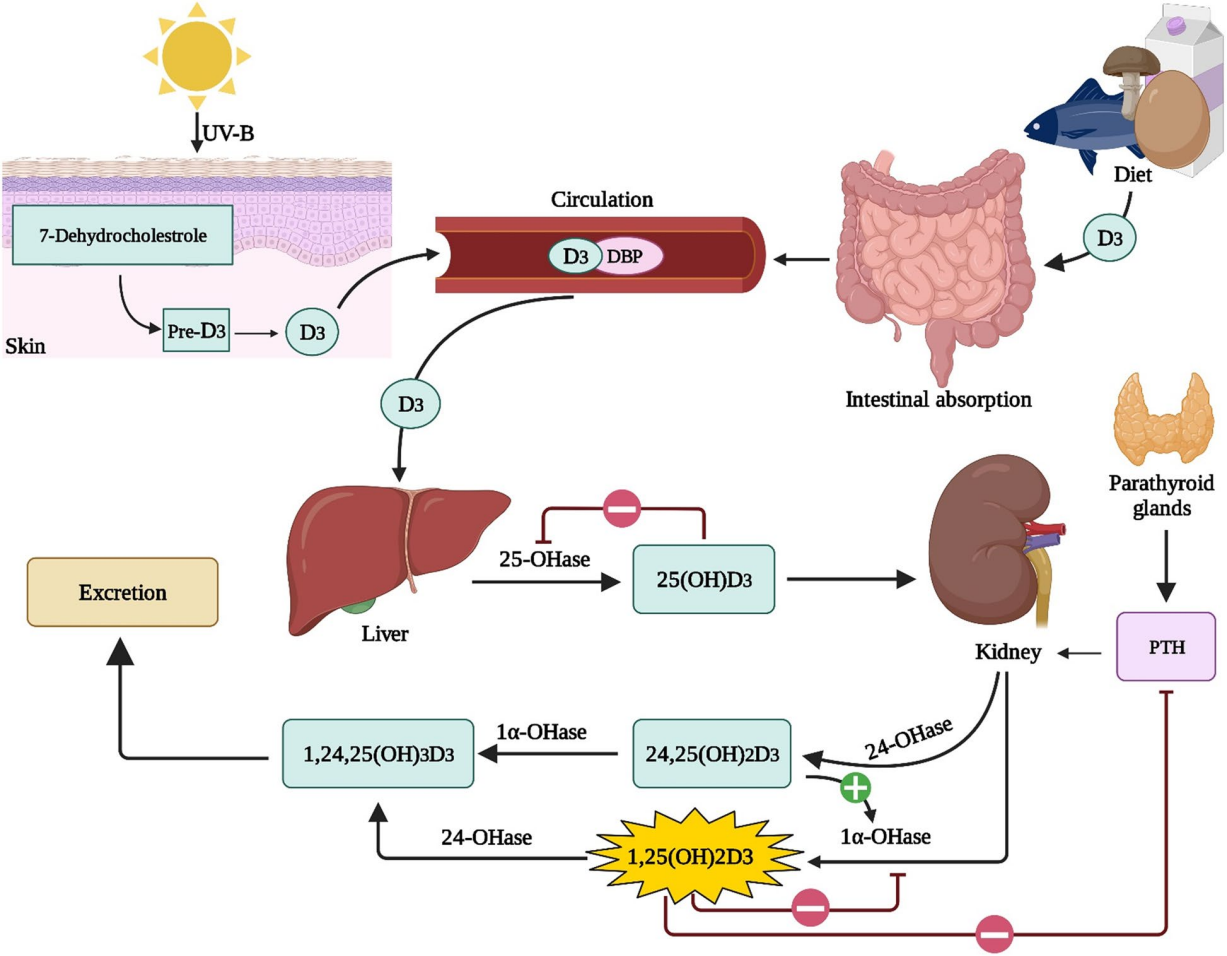
Vitamin D synthesis. Abbreviations: DBP: Vitamin D-binding protein; PTH: Parathyroid hormone

Vitamin D3, also known as cholecalciferol, is produced from 7-dehydrocholesterol in the skin by exposure to UVB sunlight [29]. Two hydroxylation steps take place in the liver and kidneys, forming 25(OH)D3 and 1,25(OH)2D3, respectively [30]. Calcitriol, also known as 1,25(OH)2D3, is the hormone that is active in the body. The first step of hydroxylation of vitamin D occurs in the liver, with the main enzyme being 25-hydroxylase. This process results in the production of 25(OH)D3 or calcidiol, which is considered the most reliable marker of vitamin D status. The second step of hydroxylation occurs in the kidney, with the main enzyme being 1α-hydroxylase. This step is regulated by osteocytes and parathyroid hormone (PTH) (Fig. 1) [31]. Vitamin D hydroxylation in the hepatic parenchyma is carried out by high-capacity cytochrome P450s, found in the endoplasmic reticulum or mitochondria [32]. 25(OH)D3 is the main circulating and stable form of vitamin D, bound to vitamin D-binding protein (DBP) or other members of the albumin superfamily in the blood [30, 33]. Hydroxylation of vitamin D occurs in various body locations and immune cells, demonstrating the diverse role of vitamin D [30, 33, 34]. The breakdown of both 25(OH)D3 and calcitriol is catalyzed by cytochrome P450 family 24 subfamily A1 (CYP24A1)/24-hydroxylase [35]. Moreover, CYP27B1 is found in various organs including skin, lymph nodes, colon, central nervous system, adrenal glands, pancreas, placenta, sweat glands and immune cells. CYP24A1 converts 1,25(OH)2D3 into calcitroic acid, which is functionally inactive and excreted through bile, feces, and urine to prevent toxic levels [31].

### Function

**Fig. 2:**
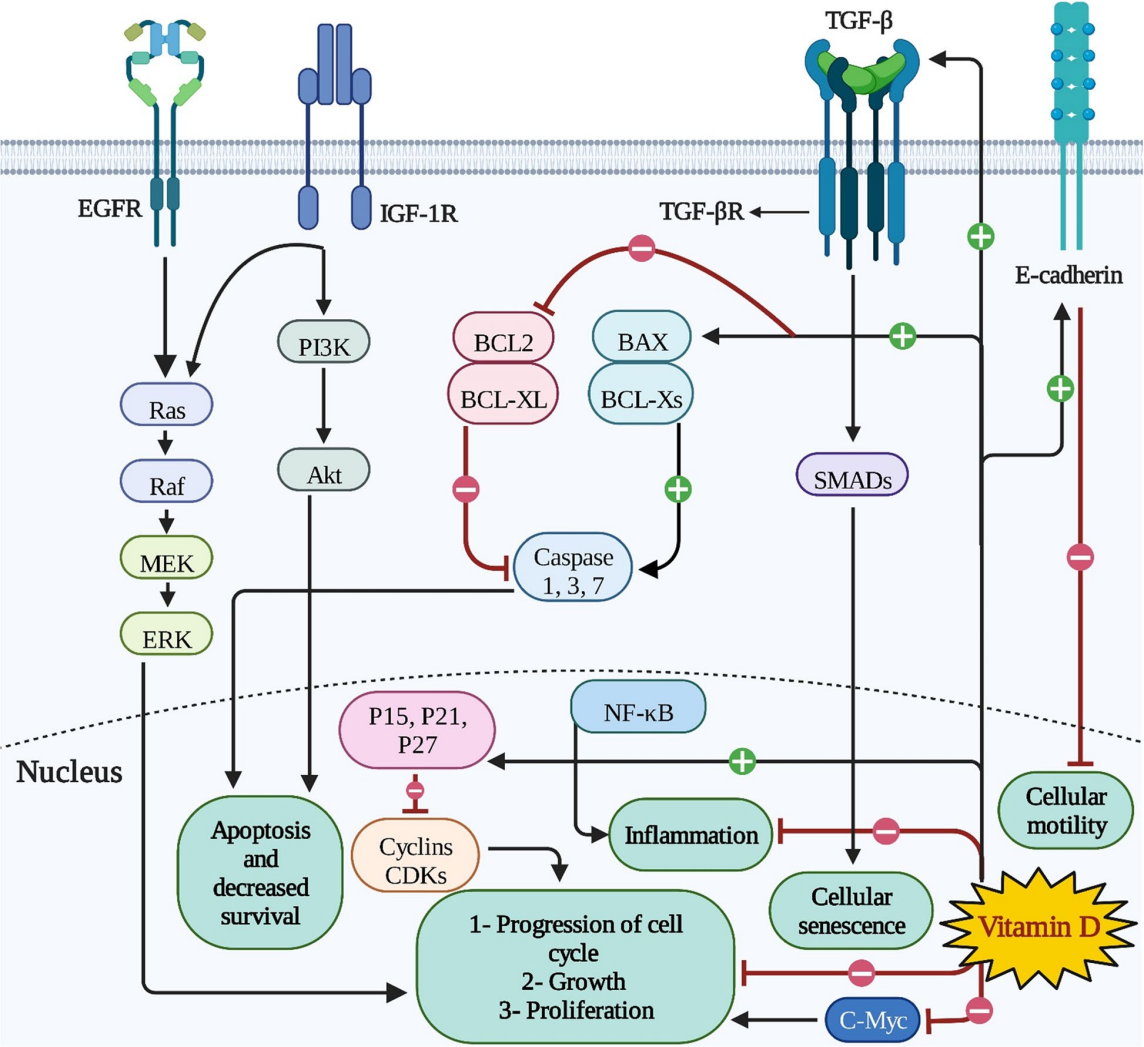
Key signaling pathways involved in endometriosis progression and the effects of vitamin D on these pathways. Abbreviations: TGF-βR: Transforming growth factor beta receptor; IGF-1R: Insulin-like growth factor 1 receptor; EGFR: Epidermal growth factor receptor

Vitamin D plays both classic and non-classic roles in the human body under normal conditions: the classic functions of vitamin D are primarily related to maintaining calcium and phosphate homeostasis, which is crucial for bone health. It enhances the intestinal absorption of these minerals, thereby preventing bone diseases like rickets in children and osteomalacia in adults. It specifically impacts calcium and phosphorus absorption in the intestines, osteoclast differentiation, calcium reabsorption from bone, and bone matrix mineralization [30, 36]. The non-classic functions of vitamin D extend beyond bone health. Vitamin D is of utmost importance in cellular proliferation, cellular differentiation, and inhibiting angiogenesis. It has been also found to play a role in immune modulation, where it helps in downregulating pro-inflammatory cytokines, such as tumor-necrosis factor alpha (TNF-α), interleukin 1 (IL-1), and IL-6, and upregulating anti-inflammatory ones. VDRs are present in many tissues, suggesting its potential role in cardiovascular health, cell differentiation, and insulin secretion [31]. The VDR is also found in various organs in the body, including the gut, bone, kidney, brain, heart, stomach, and ovaries [33, 37]. Vitamin D induces apoptosis through several mechanisms, including the insulin-like growth factor (IGFR1) - phosphatidylinositol 3-kinase (PI3K) - Akt-dependent signaling pathway, inhibiting telomerase, downregulating BCL2, inducing BAX, and activating caspase cleavage (Fig. 2).

### Dietary recommendations and sources

According to the Institute of Medicine (IOM) recommendations, individuals aged 5–64 years should consume 400–800 IU/day of vitamin D to achieve the optimal level of 50–75 nmol/L [38]. There are multiple ways to ensure adequate daily intake of vitamin D, including the synthesis of vitamin D from sun exposure, taking dietary supplements, and consuming foods rich in vitamin D [39]. However, the primary source of vitamin D is its endogenous synthesis, and dietary sources are responsible for providing less than 20% of circulating levels of vitamin D [40, 41]. Vitamin D can be found in various foods such as fatty fish, mushrooms, fish liver oils, cheese, beef liver, eggs, dark chocolate, and fortified foods like milk, yogurt, and orange juice [39].

### Etiology of Hypovitaminosis and related disorders

Vitamin D deficiency is a significant health concern, with a recommended serum level of 20 ng/ml for maintaining calcium balance and 30 ng/ml for optimal cellular health [33]. It impacts the absorption of calcium and phosphorous in the intestines, leading to issues such as impaired bone health, rickets in children, osteomalacia, osteoporosis, and an increased risk of fractures in adults. Additionally, it can cause muscular and neurological problems like tetany and seizures [30, 33]. Chronic vitamin D deficiency is associated with an increased risk of various serious health conditions, including cardiovascular disease, autoimmune diseases, inflammatory bowel disease, and certain types of cancer [33, 42].

## Vitamin D and reproduction physiology

Studies have reported direct or indirect roles of vitamin D on gonadal and reproductive functions [43–45]. The VDR is present in the male reproductive tissues of animal models, including seminiferous tubules, spermatogonia, Sertoli cells, spermatocytes, epithelial cells of the epididymis, seminal vesicles, and prostate [43]. Furthermore, according to human studies, the VDR is present in sperm [44], seminal vesicles, spermatids, vesicles within the caput epididymis, and glandular epithelium of the cauda epididymis, seminal vesicle, and prostate [45]. Although the exact mechanisms by which calcitriol influences reproductive function may remain uncertain, it applies its effects on reproductive organs through its receptor [46].

The importance of vitamin D in the male reproductive process has been emphasized due to the presence of enzymes in the testis and spermatozoa responsible for metabolizing vitamin D [47, 48]. Sood et al. studied how vitamin D deficiency affects spermatogenesis in rats and discovered that testicular glutamyl transpeptidase activity levels were reduced in rats with vitamin D deficiency, suggesting it could indicate impaired Sertoli cell function [49]. A study found that vitamin D levels were significantly lower in infertile men, indicating the important role of vitamin D in maintaining male reproductive system health by affecting factors such as spermatogenesis, sperm motility, and hormonal regulation [50, 51]. The forkhead box O (FOXO) and hypoxia-inducible factor 1 (HIF-1) pathways could play a role in explaining how vitamin D affects male infertility and the strong connections observed between them [47]. Vitamin D’s relationship with CYP24A enzyme and cAMP/PKA pathways is known to be important for spermatogenesis and sperm motility in the male genital system, but the specific mechanism of action is still not fully understood, leading to several unanswered questions in the field [52].

VDR is present in various female reproductive tissues of animal models, such as ovarian follicles, granulosa cells, follicular thecal cells, ovarian stroma, germinal epithelium, corpus luteal cells, fallopian tube epithelium, and the uterus [43]. Vienonen et al. observed the expression of VDR in the human endometrium and myometrium [53]. Similarly, our research has confirmed VDR expression in the endometrium [54]. Furthermore, the endometrium expresses the CYP27B1 gene, which encodes 1-α-hydroxylase, responsible for the local conversion of 25(OH)D3 to 1,25(OH)2D3 [55]. Additionally, the expression of CYP2R1 and CYP27A1, encoding 25-hydroxylase, has been detected in the endometrial tissue [56]. Vitamin D may impact P450 aromatase activity in granulosa cells, essential for the conversion of androstenedione to estradiol [57]. Vitamin D may regulate calcium transport across the fallopian tube epithelium, which is necessary for successful fertilization in the fallopian tube lumen [43, 58].

Vitamin D is important for the process of endometrial decidualization, where cells in the uterus transform to support embryo implantation and placental development [59]. Vitamin D plays a crucial role in embryo implantation by increasing the expression of HOXA10 in the endometrium, which helps with successful implantation and immune tolerance [60]. The presence of CYP27B1 and VDR in placental tissues indicates vitamin D’s involvement in the expression and secretion of human chorionic gonadotropin [61] and placental lactogen [62]. Vitamin D also plays a crucial role in female estrogen biosynthesis, triggering the production of progesterone, estradiol, and estrone in vitro [63].

## Reproductive disorders associated with a lack of vitamin D

### Male infertility

The deleterious impact of insufficient vitamin D levels on spermatogenesis and testicular development has been shown in animal studies [49, 64]. Vitamin D deficiency results in a significant reduction in testicular and epididymal sperm count, as well as testicular glutamyl transpeptidase activity, which is an index of Sertoli cell function [49]. Additionally, Leydig cell count reduction and degenerative alterations in the germinal epithelium are observed in vitamin D-deficient rats [49]. Men with oligospermia, asthenospermia, and azoospermia have lower calcitriol levels compared to fertile men [65]. Serum calcitriol levels are directly associated with progressive motility and total sperm count in infertile males [65]. In a cross-sectional study, infertile men with sufficient vitamin D levels showed higher semen volume, sperm counts and motility, normal sperm morphology, testosterone concentration, and testosterone/estradiol ratio compared to those with insufficient levels [66]. Clinical trials on infertile men with oligoasthenozoospermia and vitamin D deficiency demonstrated that vitamin D supplementation significantly improved sperm count and motility [67]. Another trial on infertile males with asthenozoospermia and vitamin D deficiency showed the positive effect of vitamin D supplementation on sperm motility [68]. Vitamin D deficiency is associated with an increased rate of hypogonadism [69]. However, a randomized clinical trial concluded that supplementation with vitamin D and calcium in infertile males with with vitamin D deficiency does not improve semen quality [70].

The mechanisms through which vitamin D deficiency may lead to infertility are thought to be indirect, involving extracellular calcium and phosphorus, as demonstrated in an animal study [71]. Clinical trials suggest that supplementing vitamin D in infertile males could potentially enhance sperm parameters and endocrine factors by improving oxidative stress status [72, 73]. Germ cell proliferation plays a crucial role in testicular development and spermatogenesis, with calcitriol regulating this process through VDR signaling [74–77]. Androgens, essential for germ cell differentiation and normal testicular development, may be impacted by vitamin D deficiency, affecting spermatogenesis [64, 78–80]. Enzymes like StAR, 17β-HSD, 3β-HSD, CYP11A1, and CYP17A1 are crucial for androgen synthesis in Leydig cells, with calcitriol shown to up-regulate CYP11A1 and CYP17A1 and increase 3β-HSD levels; conversely, vitamin D deficiency can decrease StAR, 3β-HSD, CYP11A1, and CYP17A1 expression in the testes [64, 81–83].

### Female infertility

Vitamin D deficiency disrupts female reproduction through a diminished probability of pregnancy, as shown in female rats [84, 85]. The impaired reproduction in females with vitamin D deficiency is proposed to be due to defective estrogen signaling [86, 87]. Female mice lacking VDR have shown hypergonadotropic hypogonadism and reduced aromatase activity, which is necessary for estrogen synthesis [88]. The aromatase null female mouse exhibits large hemorrhagic ovarian cysts, suggesting an ovulatory defect [89, 90]. VDR null mice have also shown uterine hypoplasia and are responsive to estrogen priming, indicating the role of hypogonadism in uterine defects [91]. Based on a meta-analysis, a moderate daily intake of vitamin D supplements may enhance the chances of clinical pregnancy in infertile women and can have a positive impact on pregnancy outcomes [92].

Vitamin D deficiency has been associated with a reduced rate of pregnancy in women undergoing IVF [93, 94]. The impact of vitamin D is likely through the endometrium, as no association was found between deficiency and ovarian stimulation parameters or embryo quality markers [93]. Conversely, another study found no association between serum and follicular levels of vitamin D and the pregnancy rate in the IVF cycle [95]. Additionally, vitamin D deficiency has been shown to be linked to a reduced probability of live birth after IVF or intracytoplasmic sperm injection [96]. Vitamin D treatment has been shown to reduce cytokine production in endometrial stromal cells from patients with repeated implantation failure [97].

### Polycystic ovary syndrome (PCOS)

PCOS is a prevalent endocrine disorder characterized by excessive ovarian androgen production, leading to hyperandrogenism manifested by hirsutism [98, 99]. Additionally, PCOS is marked by metabolic dysfunctions such as insulin resistance and dyslipidemia [100] and has been associated with dietary patterns [101, 102]. Studies have shown an association between vitamin D deficiency and PCOS-related metabolic abnormalities, including insulin resistance and obesity [103]. Supplementation with vitamin D in deficient PCOS patients has been shown to decrease insulin resistance and androgen levels [104]. Vitamin D may play a role in insulin receptor expression [105, 106]. VDR gene polymorphisms have also been linked to PCOS development [107]. Vitamin D status has indirect associations with sex hormone-binding globulin, the free androgen index, and anti-müllerian hormone levels [103, 108]. Correcting vitamin D status may induce endometrial proliferation in patients with PCOS during the intrauterine insemination cycle [109], suggesting potential benefits of vitamin D supplementation on metabolic disturbances in women with PCOS.

### Bacterial vaginosis

Bacterial vaginosis (BV) is a prevalent vaginal infection characterized by a decrease in Lactobacillus species and an increase in anaerobic species [110]. Our findings have confirmed that dietary patterns may play a crucial role in BV odds [111–115]. However, food items are a poor source of vitamin D, and previous studies have shown that dietary intake of vitamin D is not associated with BV prevalence [116]. On the other hand, another study has demonstrated lower levels of serum 25(OH)D3 in BV patients, indicating an indirect association between BV prevalence and vitamin D status [117]. Supplementation with vitamin D did not result in reduced BV recurrence despite a significant increase in serum 25(OH)D levels [118]. Insufficient levels of vitamin D negatively impact the ability of systemic macrophages to induce the antimicrobial peptide cathelicidin via toll-like receptors (TLRs), making the host more vulnerable to infections [119].

### Pathologies possibly aligned with vitamin D deficiency

#### Preeclampsia

Vitamin D may affect preeclampsia by regulating calcium concentration or through other mechanisms not related to calcium levels [120]. The exact mechanism underlying the role of vitamin D in preeclampsia is not well known. It is possible that vitamin D is directly or indirectly associated with the pathogenesis of preeclampsia by affecting immune function, placental implantation, angiogenesis, inflammation, and hypertension [121, 122]. Further studies are needed to specify the role of vitamin D in preeclampsia.

#### Gestational diabetes mellitus

Gestational diabetes is a type of glucose intolerance diagnosed for the first time during pregnancy [123]. Extensive research has shown that vitamin D deficiency is associated with a higher rate of gestational diabetes; however, some other studies have not shown any association. This controversy may be due to other risk factors for gestational diabetes, including obesity and lifestyle [120]. Administration of high doses of vitamin D has been observed to increase insulin sensitivity and decrease insulin levels in women with gestational diabetes [124]. It was found that vitamin D levels were lower in women with gestational diabetes compared to healthy women [125]. Vitamin D deficiency in women with gestational diabetes may increase the risk of neonatal hypoglycemia and being small for gestational age [126].

#### Preterm labor and cesarean section

The impact of vitamin D on preterm labor may be attributed to its immunomodulatory roles [127, 128]. Specifically, vitamin D plays a role in promoting innate immune responses in monocytes by stimulating antimicrobial activity [129]. A deficiency in vitamin D can lead to an increased susceptibility to infection because it impairs TLR pathways and reduces the production of cathelicidin by macrophages [127, 128]. Studies have reported an indirect association between vitamin D levels and the risk of cesarean section, which may be due to the role of vitamin D deficiency in lower calcium absorption. This deficiency can result in reduced pelvic muscle strength and prolonged labor [130].

### Reproductive malignancies

Genetically reduced levels of 25(OH)D3 are associated with increased susceptibility to ovarian cancer [131]. VDR polymorphisms are linked to the risk of cervical cancer [132] and can impact ovarian cancer susceptibility [133]. Sunlight, as a source of vitamin D, may protect against ovarian cancer-related mortality [134]. Vitamin D analogs could be potential preventive agents or therapies for cervical carcinomas, breast cancer, and ovarian cancer [135]. However, VDR protein expression may not be a prognostic factor in cervical cancer [136]. Vitamin D can regulate normal and malignant prostate cell growth and differentiation through VDR [137]. It is involved in regulating cell processes such as proliferation, differentiation, and apoptosis [138]. VDR can interact with cell cycle regulatory proteins like p21 and p27, leading to G1 arrest, and regulate cell growth factors such as c-myc and c-fos, while inducing apoptosis by down-regulating Bcl-2 [139]. VDR may inhibit androgen receptor expression, commonly found in ovarian tumors, and act as an antagonist for growth-promoting androgens in ovarian cancer cells [140].

## Endometriosis

Endometriosis is a medical condition that is distinguished by the existence of endometrial tissue in areas beyond the uterus, resulting in chronic inflammation and leading to pelvic pain and infertility. It is important to recognize the significant impact that this disease can have on a woman’s overall well-being and quality of life, as well as the substantial economic burden it can represent. As such, endometriosis should be considered a public health issue that requires special attention and resources for effective management and treatment [15, 16, 18, 141]. Endometriosis is a diverse condition that can present in three distinct forms or phenotypes: superficial peritoneal lesions (SUP), ovarian endometriomas (OMA), and deep infiltrating endometriosis (DIE). These different types of endometriosis can have varying symptoms and require different approaches to diagnosis and treatment [18]. Around 5–10% of women who are of reproductive age are affected by endometriosis, with the highest prevalence occurring between the ages of 25 and 35. The most frequent ways in which endometriosis manifests itself are related to pain and include chronic pelvic pain, dysmenorrhoea, dyspareunia, dysuria, and dyschezia [18]. Overall, risk factors for endometriosis have been identified, including menstrual and reproductive history, anthropometry, environmental exposures, and diet [142]. Specifically, risk factors for endometriosis include never giving birth, starting period at an early age, going through menopause at an older age, short menstrual cycles, heavy menstrual periods lasting longer than seven days, and higher levels of estrogen [142]. However, body mass index (BMI) assessment might not an appropriate approach to predict anthropometry instead of fat mass and/or fat-free mass measurement [143], and as our investigation has attested previously, patients with endometriosis may have lower BMI than healthy controls [144]. There is a possibility that women who have endometriosis are at an increased risk of developing various chronic illnesses, including cancer [145], cardiovascular diseases [146], adenomyosis [147], and autoimmune diseases [148]. The definite origin and pathophysiology of endometriosis are enigmatic. The hypothesizes of the origins of endometrial cells at ectopic sites include (1) retrograde menstruation, (2) metaplasia of the coelom, (3) vascular and lymphatic metastatic spread, and (4) neonatal uterine bleeding, (5) hormonal basis, (6) oxidative stress and inflammation, (7) immune dysfunction, (8) apoptosis suppression and endometrial cell fate, (9) genetic basis, (10) existence of stem cells [18, 149]. The Sampson’s retrograde menstruation theory is the oldest and well-accepted explanation for how endometriosis develops [149].

Laparoscopy is considered the most reliable method for diagnosing endometriosis. In addition to its diagnostic purposes, this medical procedure can also be used to surgically remove affected tissue, which can help reduce the pain associated with endometriosis [150]. It is advisable to ensure that a comprehensive medical history is taken and non-invasive diagnostic techniques, such as ultrasound and magnetic resonance imaging, are utilized before performing a laparoscopy [151]. According to the guidelines, treatment strategies for endometriosis are including surgical approaches, hormone therapies such as progestin and dienogest, gonadotropin-releasing hormones (GnRH) agonists/antagonists, and aromatase inhibitors. Furthermore, novel treatment approaches based on immunomodulators such as vitamin D and anti-angiogenic agents have been appraised as endometriosis treatment [152].

## The role of vitamin D in the pathogenesis of endometriosis

The role of vitamin D as an anti-inflammatory, immunomodulatory, and growth inhibitor agent for the treatment of endometriotic lesions has come under increasing scrutiny in recent years in contexts of cellular and molecular, animal, and human clinical studies (Tables 1 and 2). This concept may be further explained by a study demonstrating that endometriosis is more likely to develop in those who have low levels of vitamin D [153]. However, there is no consensus in this regard [154], and the precise role of vitamin D on the pathogenesis of endometriosis has not been absolutely discerned.

**Table 1:** Summary of the clinical trials on vitamin D supplementation in patients with endometriosis

Author, year	Study design	Region	Participants, *n*	Age, year	Intervention (form)	Duration	Outcomes		Reference
							pelvic pain	dysmenorrhea	
Almassinokiani et al., 2016	randomized, double-blind, placebo-controlled trial	Iran	Intervention: 19 Placebo: 20	Intervention: 30.84 ± 5.79 Placebo: 28.95 ± 4.71	vitamin D (50 000 IU weekly)	12 weeks	*P*-value = 0.24^*^	*p* = 0.45^*^	[155]
Nodler et al., 2020	randomized, double-blind, placebo-controlled trial	USA	Intervention: 27 Placebo: 22	Intervention: 20.0 ± 2.7 Placebo: 20.1 ± 3.5	vitamin D (2000 IU daily)	6 months	*P*-value = 0.91^*^		[156]
Mehdizadehkashi et al., 2021	randomized, double-blind, placebo-controlled trial	Iran	Intervention: 25 Placebo: 25	Intervention: 35.6 ± 7.0 Placebo: 34.8 ± 7.1	vitamin D (50 000 IU each 2 weeks)	12 weeks	*P*-value = 0.03^*^		[157]

^*^Mean difference between intervention and placebo group

**Table 2:** Summary of the clinical trials on vitamin D supplementation in patients with endometriosis using experimental assays

Author, year	Study design	Region	Intervention (form)	Duration	Outcomes					Reference
					expression level of CD44s in the eutopic endometrium	expression level of CD44v in the eutopic endometrium	expression level of CD44v6 in the eutopic endometrium	concentration of soluble CD44 in the endometrial fluid	expression of active form of b-catenin	
Pazhohan et al., 2018	a randomized exploratory trial	Iran	vitamin D (50 000 IU weekly)	12–14 weeks	decreased after modification of the circulating levels of 25(OH)D	decreased after modification of the circulating levels of 25(OH)D	decreased after modification of the circulating levels of 25(OH)D	decreased after modification of the circulating levels of 25(OH)D		[158]
Pazhohan et al., 2020	a randomized exploratory trial	Iran	vitamin D (50 000 IU weekly)	12–14 weeks					*P*-value = 0.012	[159]

Endometriosis is a disease that mimics a pre-cancerous state and fulfills the hallmarks of cancer. Also, endometriosis may increase the risk for autoimmune conditions. Given that vitamin D is an agent with anti-proliferative, anti-inflammatory, and immunomodulatory features, thus there is a hypothesized link between endometriosis and vitamin D [28, 153, 160, 161]. Endometriosis exhibits many characteristics of an autoimmune disorder, and it has been suggested that an immune-mediated deficiency in the identification and clearance of endometrial fragments refluxed in the peritoneal cavity plays a paramount role in the advancement of endometriosis [162, 163]. Lymphocytes, macrophages, and dendritic cells that are in an activated state show a significant presence of VDR and metabolizing enzymes, including 1-α hydroxylase and 24-hydroxylase [164], suggesting that vitamin D can be generated within the immune system itself, and it functions in an autocrine-paracrine manner. In the following sections, we will provide pathophysiology and evidence demonstrating the importance and role of vitamin D in the pathogenesis of endometriosis.

### Pathophysiology of endometriosis

**Fig. 3:**
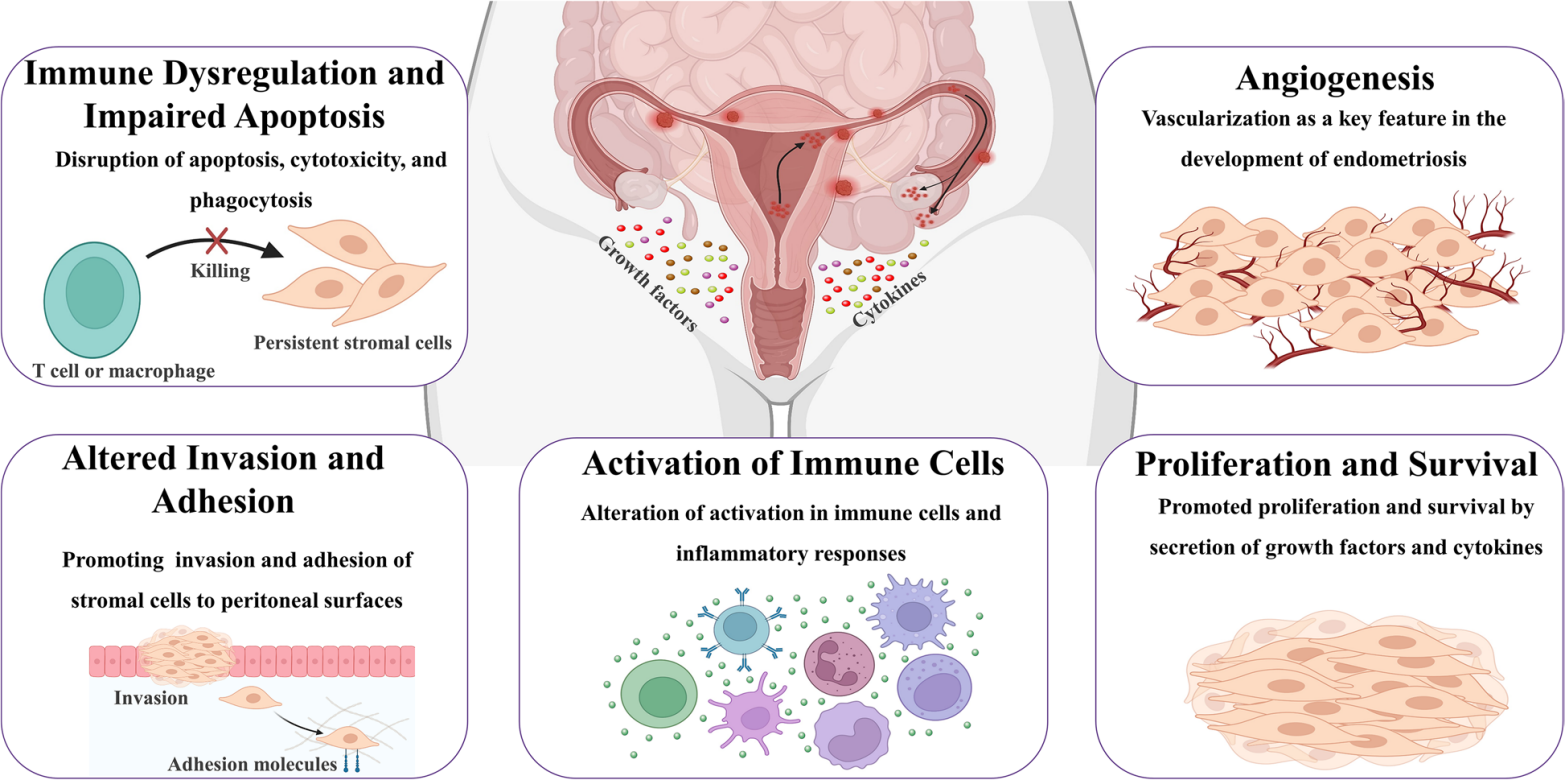
The causes and mechanisms underlying the development of endometriosis

As per the theory of Sampson’s retrograde menstruation, functional tissue from the endometrium can be carried through the fallopian tubes, possibly due to imbalanced uterine contractions. Upon reaching the peritoneal cavity, these fragments may attach themselves, grow, and infiltrate surrounding pelvic structures [149, 165]. As earlier was mentioned, the primary theories of the origins of endometrial cells at the locations of ectopic endometriosis include retrograde menstruation, metaplasia of the coelom, vascular and lymphatic metastatic spread, and neonatal uterine bleeding. However, the exact origin and pathophysiology of the condition remain unknown. However, there are several tentative explanation, which may contribute to establish and progress endometriosis (Fig. 3). In this section, other factors that orchestrate cell survival, proliferation, and ectopic lesion formation and maintenance are highlighted.

#### Altered steroid biosynthesis and receptor response

Oestrogens are crucial for the growth of endometrial cells, and environmental factors like pesticides and toxicants may contribute to abnormal cell development in women with endometriosis by impacting the production and breakdown of oestradiol [166]. An oestrogenic microenvironment is established close to endometriotic lesions due to the local accumulation of oestradiol [167]. A network of genes including greb1, myc, and ccnd1 that govern cell mitogenesis is activated when there are high local concentrations of oestradiol and overexpression of estrogen receptor alpha (ERα) and ERβ [168]. The development of ERβ function contributes to the progression, establishment, and invasion of endometriotic tissues through inhibiting apoptosis induced by TNF-α, increasing IL-1β, and enhancing epithelial-mesenchymal transition (EMT) signaling [169].

#### Increased invasiveness and vascularization

Vascularization is a key feature in endometriosis development, with studies suggesting that processes like angiogenesis, vasculogenesis, and inosculation play a role in vascularizing endometriotic lesions [170]. Angiogenesis is the creation of new blood vessels, triggered by growth factors like vascular endothelial growth factor (VEGF). Studies have looked at VEGF expression in endometriosis models and patient samples [170]. In addition to VEGF, there are multiple other factors that have been documented as facilitators for angiogenesis in cases of endometriosis. These include fibroblast growth factor (FGF), platelet-derived endothelial cell growth factor (PD-ECGF), angiopoietin (Ang)-1/2, MMP-1/2/9, endoglin, activin A, galectin-1, cofilin-1, microsomal prostaglandin E synthase (mPGES)-1, macrophage migration inhibitory factor (MIF), IL-1β, IL-4, IL-17 A, prostaglandin F2alpha (PGF2α), and synuclein-γ [170]. In endometriotic stromal cells (ESCs), the over-activation of the AKT signaling pathway is another cause of invasiveness and establishment [171]. Macrophages are capable of producing trophic factors and contributing to the process of angiogenesis. As a result, they make a contribution to the progression of endometriotic lesions [172].

#### Altered inflammatory responses

Endometriosis is characterized by inflammation due to the presence of ectopic tissue in the peritoneal cavity leading to increased synthesis of prostaglandins, cytokines, and chemokines [173, 174]. Patients with endometriosis have a greater number of circulating regulatory T cells compared to those without the disease, which could be observed as a way for the body to control the inflammation present in this condition [175]. Endometriosis has been linked to two significant types of chemokines. Eosinophils, monocytes, and T lymphocytes are the primary targets of CC-chemokine ligands, including CCL5, CCL2 and CCL11. Neutrophils and monocytes are recruited by CXC-chemokine ligands, including CXCL1, CXCL8, CXCL5 and CXCL12 [176]. A variety of adhesion molecules, growth factors, and pro-inflammatory cytokines are secreted by activated macrophages and ESCs into the peritoneal fluid (PF) and the milieu of endometriosis lesions, which include fibronectin [177], intercellular adhesion molecule 1 (ICAM1) [176], insulin-like growth factor-1 (IGF-1) [178, 179], monocyte chemoattractant protein-1 (MCP-1) [17, 178], hepatocyte growth factor (HGF) [178–180], IL-1 [176], IL-6 [17, 181], IL-8 [17, 181], CCL5 [17], platelet-derived growth factor (PDGF) [182], epidermal growth factor (EGF) [182], VEGF [165, 180], monocyte/macrophage-derived growth factor (MDGF) [182], TNF [183], and MMP-9 [165]. Nuclear factor-κB (NF-κB) is activated in peritoneal endometriotic lesions, most likely owing to an increase in the amounts of pro-inflammatory mediators and cytokines found in the milieu of the lesion. It has been proven that ESCs and peritoneal macrophages obtained from women who have endometrioma both upregulate NF-κB [15]. Iron accumulation in endometriotic lesions from in-situ menstruation can lead to the production of reactive oxygen species (ROS), which increases NF-κB activity in endometriotic stromal cells [184].

#### Impaired apoptosis

Apoptosis is disrupted in endometrial cells in women with endometriosis, causing them to continue developing in ectopic locations. Apoptosis levels in the normal endometrium of patients with endometriosis were found to be reduced compared to those without the condition. Additionally, the endometrial tissue in abnormal locations of patients with endometriosis showed even lower levels of apoptosis when compared to their normal endometrial tissue [185–187]. Fas ligand (FasL)-expressing cells engage with immune cells expressing the Fas molecule, inducing apoptosis [188]. Metalloproteases can cleave membrane-bound FasL, producing soluble FasL. Immune cells expressing the Fas protein may undergo apoptosis when exposed to FasL in the peritoneal cavity of women with endometriosis [162].

### Cellular and molecular studies

Recently, our current evidence demonstrated the downregulation of proliferation and protein expression of MCP-1, HGF, and IGF-1 –which are involved in proliferation, invasion, and angiogenesis, respectively– in PBMCs, and PFMCs of endometriotic women upon vitamin D treatment. Additionally, this treatment remarkably reduced MCP-1, HGF, and IGF-1 expression at the gene and protein levels in EESCs and EuESCs [178]. We found that vitamin D boosts cell adhesion and reduces invasion and proliferation of endometrial stromal cells in both ectopic and eutopic endometriotic cells by inhibiting the production of IL-6, Bcl-2, Bcl-xL, and VEGF-α [28]. A study found that levels of CD44 protein, which is involved in cell adhesion, were significantly reduced in endometrial cells of women with endometriosis after they were given oral vitamin D [158]. In another empirical study, we indicated that endometriosis is characterized by an upregulation in the expression of the PDGFB and EGF at the gene level. Notably, we showed that vitamin D3 significantly reduced PDGFB and EGF gene expression, indicating that vitamin D supplementation might be a therapeutic approach to managing endometriosis [182]. Finally, another study by our group explored the effect of vitamin D3 on biological behavior of the EuESCs and EESCs derived from women with endometriosis. Overall, our findings proved that vitamin D3 modulates characteristics of human ESCs in association with endometriosis [28]. As a proof-of-concept work, Miyashita et al. examined the in vitro effects of vitamin D3 on inflammatory immune responses and the proliferation of ESCs from women with endometriosis, and the serum levels of this vitamin were also investigated in these patients. The study stated that endometriosis was related to a decreased vitamin D status, and this vitamin considerably reduced viable ESC numbers, DNA synthesis, and the expression of IL-1β, IL-8, prostaglandins, MMP-2, and MMP-9, finally leading to reduced inflammation and proliferation in endometriotic cells [161]. Ingles et al. used high-throughput RNA sequencing to investigate the effect of vitamin D3 on numerous genes expression in an endometriosis stromal cell line. They found that vitamin D3 reduced pathways related to angiogenesis, invasion, and cellular motility [189]. Recently, we conducted a study to determine the effects of vitamin D on proliferation, cell cycle, and apoptosis of ESCs. Our results for the first time indicated that vitamin D has a growth-inhibiting and pro-apoptotic effect on ESCs from women with endometriosis [190].

Mechanistically, vitamin D can reduce inflammation, proliferation, cytokine production, and growth factors expression through several ways, explaining its beneficial effects for reliefing endometriotic lesions of patients with endometriosis. First, vitamin D inhibits NF-κB activation via promoting the stability of inhibitor of kappa B alpha (IkBα) protein [191]. Second, this vitamin can up-regulate the expression of mitogen-activated protein kinase phosphatase-1 (MKP-1). MKP-1 is known to preferentially inactivate p38 and c-Jun N-terminal kinase (JNK), leading to subsequent inhibition of pro-inflammatory cytokines biosynthesis [192]. Third, in the context of cancer setting, vitamin D has been shown to suppress the proliferation of liver cell lines by down-regulating c-Met and extracellular signal-regulated kinase (ERK) expression [193]. Another mechanism suppressing the development of endometriosis is that vitamin D may negatively interfere with IGF-1R/phosphatidylinositol 3-kinase (PI3K)/AKT signaling pathway. Indeed, our previous study revealed that vitamin D down-regulates IGF-1 expression [27]. A tentative mechanism for vitamin D-induced endometriotic cell cycle arrest is that vitamin D may act as a regulator of cyclin and cyclin-dependent kinase (CDK). For illustration, evidence demonstrated vitamin D treatment on cancer cells promoted the expression of CDK inhibitors P21 and P27, and it down-regulated cyclin D and CDKs 4 and 5, resulting in cell cycle arrest in G0/G1 phase and inhibition of the NF-κB signaling pathway (Fig. 2) [28].

All in all, growth factors, mediators of inflammation, and immune factors are of irrefutable importance in the pathogenesis of endometriosis. The aforementioned findings accentuate that vitamin D could reduce the expression of molecules and growth factors involved in angiogenesis, proliferation, invasion, migration, and inflammation in human endometriotic cells. These in vitro studies suggest the possibility of utilizing vitamin D for therapeutic purposes in endometriosis.

### Animal studies

A study aimed at unraveling the role of vitamin D in the treatment of endometriosis in rats. Endometriotic implants were regressed when vitamin D was treated. This was significantly accomplished by increasing tissue inhibitor of metalloproteinase-2 (TIMP-2) expression, suppressing neovascularization, and modulation of MMP-9 and VEGF expression in rats treated with vitamin D [194]. Consistent with this study, the finding of another study revealed regression of endometrial implants as a result of vitamin D treatment in a rat model of endometriosis [195]. Vitamin D was found to prevent the development of endometriosis in mice by controlling interleukin-17 levels [196].

Therapeutic approaches for endometriosis have witnessed a progressive but substantial modification. In addition to vitamin D, VDR agonists also are applicable to the treatment of endometriosis. Mariani et al. have proven that elocalcitol, a selective VDR agonist, prevented the development of endometriosis and the establishment of lesions in a mouse model by preventing peritoneal inflammation and macrophage recruitment [197].

### Human clinical studies

In regard to the association between the risk of endometriosis and plasma vitamin D level, the results are controversial. Some studies indicated that there is an inverse association between levels of vitamin D in serum and the risk of endometriosis [20, 153, 161, 198, 199]. A relationship has been observed between vitamin D levels and the severity of endometriosis, with lower levels of vitamin D being associated with more severe cases of the condition. Furthermore, compared to healthy individuals, women who have endometriosis tend to have lower levels of vitamin D. Thus, a possible risk factor for endometriosis could be attributed to hypovitaminosis D [199]. An investigation discovered a high prevalence of women suffering from both hypovitaminosis D and endometriosis [200]. On the other hand, two studies reported that endometriosis has been linked to elevated levels of vitamin D in serum [201, 202], conveying a direct association. Contrary to all these studies, Agic et al. found that there is no disparity detected in the vitamin D levels in the blood of women with endometriosis and those without the condition [21]. Furthermore, the results showed that endometriosis-affected women had unaffected concentrations of DBP in their serum and PF [203]. Additionally, in an analysis, Ferrero et al. found a reduced level of DBP in the PF but not in the plasma of women with untreated endometriosis [204]. A case-control study concluded that there is no association between serum vitamin D levels and different phenotypes of endometriosis [205]. Likewise, in a study which set out to determine the association, Baek et al. found that vitamin D and DBP levels were not associated with the severity of endometriosis [206]. The evaluation of DBP and lactoferrin concentrations did not show significant differences between women with and without endometriosis. However, the correlations between these proteins in plasma and peritoneal fluid of women with endometriosis may offer a new panel of markers for identifying high-risk patients [207]. The findings of a meta-analysis suggest that dietary antioxidant supplementation, particularly with vitamin D and melatonin, may have a beneficial impact on the severity of endometriosis-related dysmenorrhea and pelvic pain [208, 209]. Overall, the conflicting findings could be attributed to variations in sample size, age groups or populations, study design and its limitations, and inclusion/exclusion criteria. In other words, the role of vitamin D in endometriosis is challenging and complex [154]. This matter prompts scientists worldwide to conduct new clinical trials and reach a consensus in the field.

The presence of VDR and the mitochondrial cytochrome P450 enzyme 25-hydroxyvitamin D3 in the uterus and immune cells proposes that vitamin D may play a role in the pathophysiology of the endometrial cells. Additionally, vitamin D decreases the synthesis of prostaglandins, which could potentially have a positive impact on the uterus and endometrial cells [210]. A clinical trial has indicated that supplementation with vitamin D resulted in substantial alterations in the level of pelvic discomfort experienced by young women who suffered from endometriosis. However, this result was not statistically different from the placebo group [156]. Another clinical trial reported that vitamin D therapy did not have a significant impact in lowering dysmenorrhea and pelvic pain in patients with endometriosis diagnosed and treated by laparoscopy [155]. Finally, an intervention study enrolled forty women with primary dysmenorrhea to assess the effect of a single-loading oral dose of vitamin D on dysmenorrhea. Participants were randomized into 2 groups, including women receiving vitamin D before the expected beginning of their next menstrual cycle (*n* = 20) and a placebo group (*n* = 20). The mean pain score decreased significantly in the experimental group [210]. Vitamin D treatment-related decreased pain may be due to reducing in prostaglandin synthesis and an increment in prostaglandin inactivation via inhibited cyclo-oxygenase 2 (COX-2) and upregulated 15-hydroxyprostaglandin dehydrogenase, respectively. Furthermore, vitamin D could exert its anti-inflammatory effects through inhibiting NF-κB and up-regulated activation of mitogen-activated protein kinase phosphatase signaling pathways (Fig. 2) [211].

### Genetic studies

Several studies aimed to address whether DBP and VDR are genetic risk factors for endometriosis‑associated infertility. To date, there has been no consensus on this issue, and discrepancies in different ethnic populations were reported. Studies revealed that variants in the VDR gene and DBP serve as genetic risk factors referring to infertility associated with endometriosis and the pathogenesis of endometriosis [212, 213]. Contrary to these findings, the results of two studies have indicated that VDR and DBP gene polymorphisms may have no role in endometriosis susceptibility [54, 214].

## Conclusion and future directions

All in all, this article aimed to clarify the significance of vitamin D in the physiological and pathological processes of reproduction in males and females. However, our streamlined approach was to address the role of vitamin D in endometriosis. Cellular and molecular studies have shown that vitamin D treatment can downregulate key molecules involved in proliferation, invasion, angiogenesis, and inflammation in endometriotic cells. Animal studies further support these findings by demonstrating regression of endometriotic implants with vitamin D treatment in rat models. Human clinical studies, however, have yielded mixed results regarding the association between vitamin D levels and the risk and severity of endometriosis. Despite these discrepancies, genetic studies have suggested that genetic variants in the VDR and DBP genes may play a role in endometriosis-associated infertility. Overall, the findings from these studies emphasize the potential of vitamin D as a therapeutic option for managing endometriosis by targeting key pathways involved in its pathogenesis. Further research is needed to fully understand the mechanisms and optimal strategies for utilizing vitamin D in the treatment of endometriosis.

The development of endometriosis is influenced by various inflammatory processes that cause both local and systemic effects in the body. Thus, inflammatory processes in endometriosis and leveraging the inflammatory process-oriented therapeutic approaches will be an attractive research direction for future studies. In this regard, a long-acting antibody directed against IL-8 (AMY109) showed potential therapeutic value in reducing the size of endometriotic lesions and extent of adhesions and fibrosis in a syngeneic model of endometriosis in cynomolgus macaques [215]. Additionally, treatment with pexidartinib decreases inflammatory signaling and cell survival in endometriosis by suppressing macrophage-colony stimulating factor 1 receptor and stem cell growth factor receptor KIT [216]. Moreover, researchers have found a possible link between Fusobacterium infection in the endometrium and endometriosis. Pre-clinical studies using a mouse model showed that treatment with metronidazole and chloramphenicol reduced the severity of the disease in infected animals [217]. All in all, a hopeful strategy for treating endometriosis might be the consideration of a multi-faceted treatment involving combination therapy of aforementioned agents with vitamin D. These approaches need to be tested to prove the synergistic effects of these agents in future studies.

The adverse effects of endometriosis on the patients’ central nervous system (CNS) is a significant issue that deserves a special attention. This may be an underlying factor that lead to anxiety, depression, and other psychological disorders in women with endometriosis. Researchers found that microglial cells in various regions of the brain were enlarged in a mouse model of endometriosis, indicating overall activation of these cells. This finding may have implications for understanding chronic pain and other neurological symptoms associated with endometriosis [218]. As the potential favorable effects of vitamin D in the context of neurological disorders [219, 220], investigating the role of vitamin D in relieving neurologic complications related to endometriosis would be an active research area.

Investigating the precise effects of vitamin D on pathogenesis of endometriosis at the transcriptome level and proteome is an unmet need that should be assessed. These investigations is a crucial component for expanding single cell profiling, which will assist in studying the development of endometriosis and discovering innovative methods for diagnosing and managing the condition. In this regard, there are some studies [221–223]. However, this topic is of paramount importance, and the field needs to move forward to take one step closer to a better understanding of personalized treatment for endometriosis.

Our review is not limited to specific age groups or populations. Thus, the overall conclusions drawn from this review might apply to a broader population. However, according to the National Institute for Health and Care Excellence (NICE) guideline, the need for vitamin D is a prioritization for specific age groups or populations. Indeed, the need to receive vitamin D is different in various age groups and populations. These groups consist of infants and children under 4 years old, pregnant and breastfeeding women, especially teenagers and young women, individuals over the age of 65, those with limited sun exposure due to cultural practices, being homebound, or spending extended periods indoors, and individuals with darker skin tones such as those of African, African-Caribbean, or South Asian descent [224, 225].

## Data Availability

No datasets were generated or analysed during the current study.

## Abbreviations

VDR: Vitamin D receptor
PCOS: Polycystic ovary syndrome
IVF: In vitro fertilization
ESC: Endometriotic stromal cell
EuESC: Eutopic endometrial stromal cell
EESC: Ectopic endometrial stromal cell
PBMC: Peripheral blood mononuclear cell
PFMC: Peritoneal fluid mononuclear cell
VEGF: Vascular endothelial growth factor
DBP: Vitamin D-binding protein
NF-κB: Nuclear factor-κB
TNF: Tumor-necrosis factor
PF: Peritoneal fluid
MMP: Matrix metalloproteinase
CDK: Cyclin-dependent kinase
BMI: Body mass index
EGF: Epidermal growth factor
PDGF: Platelet-derived growth factor
TLR: Toll-like receptor
